# The effect of abolishing instructions to fast prior to contrast-enhanced CT on the incidence of acute adverse reactions

**DOI:** 10.1186/s13244-020-00918-y

**Published:** 2020-10-23

**Authors:** Yoshito Tsushima, Yuko Seki, Takahito Nakajima, Hiromi Hirasawa, Ayako Taketomi-Takahashi, Shogo Tan, Takayuki Suto

**Affiliations:** 1 Department of Diagnostic Radiology and Nuclear Medicine, Gunma University Gdaduate School of Medicine, 3-39-22 Showa, Maebashi, Gunma 371-8511 Japan; 2 Department of Radiology, Gunma University Hospital, Maebashi, Japan

**Keywords:** Computed tomography (CT), Iodinated contrast media, Acute adverse reaction, Fasting

## Abstract

**Objectives:**

To evaluate the effect of abolishing instructions to fast prior to contrast-enhanced CT on acute adverse reactions (AARs).

**Methods:**

In our institution, we instructed patients to fast one meal before contrast-enhanced CT examinations. However, we abolished these instructions at the end of March 2019, and solid food intake was not restricted before contrast-enhanced CT after this date. The differences in the incidence of AARs before (December 2015–November 2018, *n* = 43,927) and after (April 2019–March 2020, *n* = 14,676) abolishing instructions to fast were compared. We allowed 4 months (December 2018–March 2019) for this policy change to fully permeate the CT referrals. The medical records of patients who vomited were retrospectively reviewed by one of the authors for notations of aspiration or aspiration pneumonia attributable to vomiting.

**Results:**

The overall incidence of AARs before (1.60%, *n* = 705) and after abolition (1.40%, *n* = 205) did not change significantly. As the chemotoxic reactions, the incidence of nausea decreased significantly (0.31 to 0.18%, *p* = 0.006). The incidence of vomiting did not change (0.12 to 0.16%), and there were no cases of aspiration pneumonia attributable to vomiting during the study period. The incidence of severe hypersensitivity/allergy-like reactions did not change (0.06 to 0.05%).

**Conclusions:**

Abolishing instructions to fast decreased the incidence of nausea, but did not affect the incidence of vomiting. No cases of aspiration pneumonia attributable to vomiting were found. Our study confirmed that fasting is not required prior to contrast-enhanced CT.

## Key points

- Abolishing instructions to fast prior to contrast-enhanced computed tomography (CT) decreased the incidence of nausea, but did not affect the incidence of vomiting.
- There were no cases of aspiration pneumonia attributable to vomiting, and the overall incidence of acute adverse reactions (AARs) was unchanged after abolishing instructions to fast.
- Our study confirmed that fasting is not required prior to contrast-enhanced CT.

## Introduction

Fasting prior to contrast-enhanced CT has traditionally been considered necessary due to concerns of nausea and vomiting, which are common acute adverse reactions (AARs). Emetic complications were frequent with use of ionic high-osmolar iodinated contrast media (4.58% for nausea and 1.84% for vomiting), and gastrointestinal emptying was thought to reduce the risk of not only nausea and vomiting, but also aspiration [1, 2]. Preventing emetic complications would prevent aspiration pneumonia, so instructing patients to fast prior to contrast-enhanced CT might have made sense when ionic high-osmolar iodinated contrast media were used more frequently. However, the incidence of nausea and vomiting dramatically decreased after the introduction of non-ionic low-osmolar contrast media (1.04% for nausea and 0.36% for vomiting), raising questions on the efficacy of this policy [2].

Recently, some investigators reported that the occurrence of nausea and vomiting lacked correlation with the preparative solid food status, suggesting solid food fasting was not essential [2–4]. In addition, since many patients may excessively fast fluids as well as solid food, they should be encouraged to prevent dehydration and not to excessively fast fluids, even when instructed to fast [3]. The Society of European Urogenital Radiology (ESUR) released the new guidelines (ver. 10.0) in 2018 [5] clearly stating “fasting is not recommended before administration of low- or iso-osmolar non-ionic iodine-based contrast media or of gadolinium-based agents.” In our institution, the traditional policy of fasting prior to contrast-enhanced CT examination has been abolished to follow this new statement. However, there has been no report indicating whether the policy change affects the incidence of all types of AARs, and the concern for increased aspiration or aspiration pneumonia remains.

The aim of this study was to evaluate the effect of abolishing instructions to fast prior to contrast-enhanced CT on the frequency of AARs.

## Materials and methods

For more than 40 years, patients in our institution received instructions to fast one meal (restrict solid food intake) before their contrast-enhanced CT examination: patients with CT examination in the morning did not have breakfast, and those with an examination in the afternoon did not have a lunch. We decided to change this policy to follow the ESUR guidelines ver. 10.0 released in 2018 [5], and made an in-house announcement in December 2018 that instructions to fast were no longer necessary for patients undergoing contrast-enhanced CT examinations. Fluid intake was not restricted, either before or after the announcement.

Non-ionic low-osmolar iodinated contrast media were intravenously administered with an automatic injector (Dual Shot Nemoto Kyorindo, Tokyo, Japan) using a 22G, 24G, or 20G needle. The contrast media dose was selected according to patient weight and the purpose of CT examinations, based on our institutional protocol. All contrast-enhanced CT examinations were performed with one of five contrast media: iopromide (Iopromide Injection; FUJIFILM RI Pharma, Tokyo, Japan), iomeprol (Iomeron; Eisai Co., Ltd., Tokyo, Japan), iopamidol (Iopamiron; Bayer Yakuhin, Osaka, Japan), iohexol (Omnipaque; Daiichi Sankyo Co., Ltd., Tokyo, Japan), and ioversol (Optiray; Gurbet Japan, Tokyo, Japan). During the study period, there was no major change in radiologists’ charge and no change in the protocol.

All types of AARs were recorded by both radiology technologists and/or attending radiologists in the radiological information system (RIS). Patients were instructed to stay in CT suite waiting area for approximately 15 min after examinations, and all AARs, defined as reactions that occurred before patients left the radiology department, were recorded. The data before (December 2015–November 2018, 43,927 cases; age, 64.9 ± 14.2 years [mean ± SD], 0–99 [range]) and after the policy change (April 2019–March 2020, 14,676 cases; age, 65.7 ± 14.4 [mean ± SD], 0–99 [range]) were reviewed (Table 1).

**Table 1:** Number of CT examinations during the survey period

Anatomical regions	Before the policy change		After the policy change	
	Number of CT examinations	Enhanced CT examinations (%)	Number of CT examinations	Enhanced CT examinations (%)
**Head/neck**	22,667	4797 (21.2%)	7003	1793 (26.5%)
**Chest**	13,631	1418 (10.4%)	3534	355 (9.1%)
**Abdomen**	5040	1711 (33.9%)	1746	637 (36.5%)
**Chest + abdomen**	46,945	33,434 (71.2%)	16,096	11,202 (69.6%)
**Others**	5851	2507 (42.8%)	30,611	689 (36.7%)

We allowed 4 months (transition period; December 2018–March 2019) for this policy change to fully permeate the CT referrals, and data from during this period were not used in the analysis in this study. When the referral physicians thought fasting prior to CT examinations necessary, for instance for virtual gastroscopy, CT colonoscopy, and 3D anatomic reconstruction for preoperative planning, they instructed patients to fast.

Our classification of AARs, modified from the ESUR guidelines ver. 10.0 [5], is shown in Table 2. Since urticaria, itching, and erythema often appear at the same time, in our institution, these reactions are collectively categorized as “mild allergy-like/hypersensitive AARs.”

**Table 2:** AARs before and after the policy change

	AARs		Incidence	
	Severity	Types	Before policy change (***n*** = 43,927)	After policy change (***n*** = 14,676)
**Overall**			705 (1.60%)	205 (1.40%)
**Allergy-like/hypersensitivity**	**Mild**	Urticaria/itching/erythema	313 (0.71%)	103 (0.70%)
	**Moderate**	Bronchospasm	0	0
		Facial/laryngeal edema	0	1 (0.01%)
	**Severe**	Hypotensive shock	25 (0.06%)	7 (0.05%)
		Respiratory arrest	0	0
		Cardiac arrest	0	0
**Chemotoxic**	**Mild**	Nausea	137 (0.31%)	26 (0.18%)*
		Vomiting	55 (0.12%)	23 (0.16%)
		Anxiety	175 (0.40%)	45 (0.31%)
		Vasovagal reaction which resolve spontaneously	0	0
	**Moderate**	Vasovagal reaction	0	0
	**Severe**	Arrhythmia	0	0
		Convulsion	0	0

This classification was modified from ESUR guidelines on contrast media ver. 10.0 [5]

*p = 0.006

The medical records of patients who vomited were retrospectively reviewed by one of the authors for notations of aspiration or aspiration pneumonia attributable to vomiting. This study did not set criteria for the diagnosis of aspiration pneumonia. Diagnosis was based on documentation on the patient’s chart. If there was no clear documentation of the diagnosis of aspiration pneumonia, or clinical findings suspicious for aspiration pneumonia, the patient was not considered to have aspiration pneumonia.

For statistical analyses, SPSS Statistics 25 (IBM Japan, Tokyo) was employed. Fisher’s exact test and chi-square test were used, and *p* value less than 0.05 was considered significant. This study was approved by the institutional research ethics committee, and the informed consents from patients, radiology technologists, and radiologists were waived.

## Results

The numbers of unenhanced and enhanced CT examinations during the survey period are shown in Table 1. There was no significant difference in the anatomic regions of CT examination or rates of enhanced CT before and after the policy change.

**Fig. 1:**
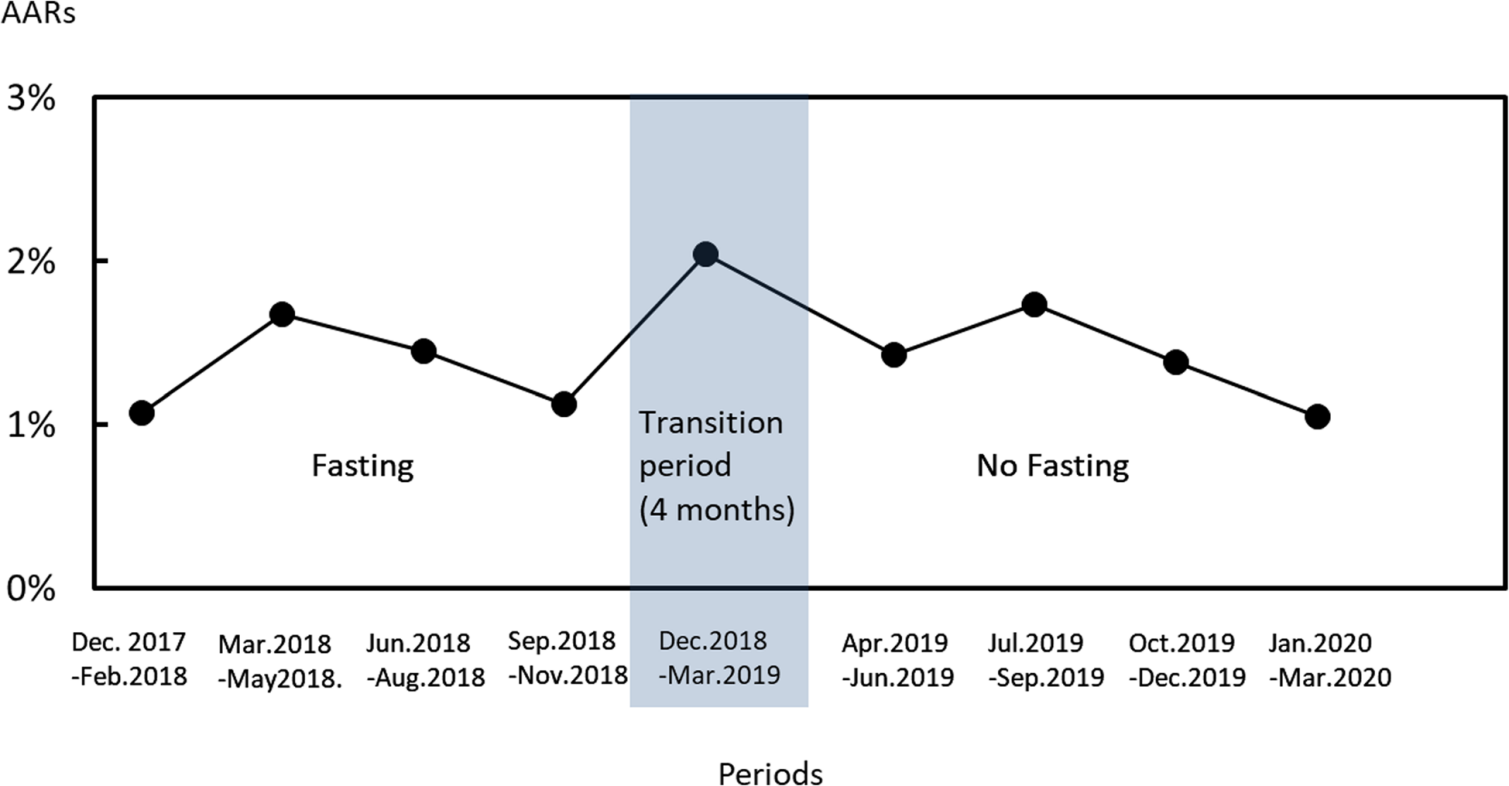
Transition of overall incidence every 3 months

There was no significant difference in the overall incidence of AARs before (1.60%, *n* = 705) and after (1.40%, *n* = 205) the policy change. Figure 1 shows the transition of the overall incidence every 3 months (the transition period was 4 months). It appeared to be slightly higher during the transition period and early after transition, but it was not a statistically significant change.

**Fig. 2:**
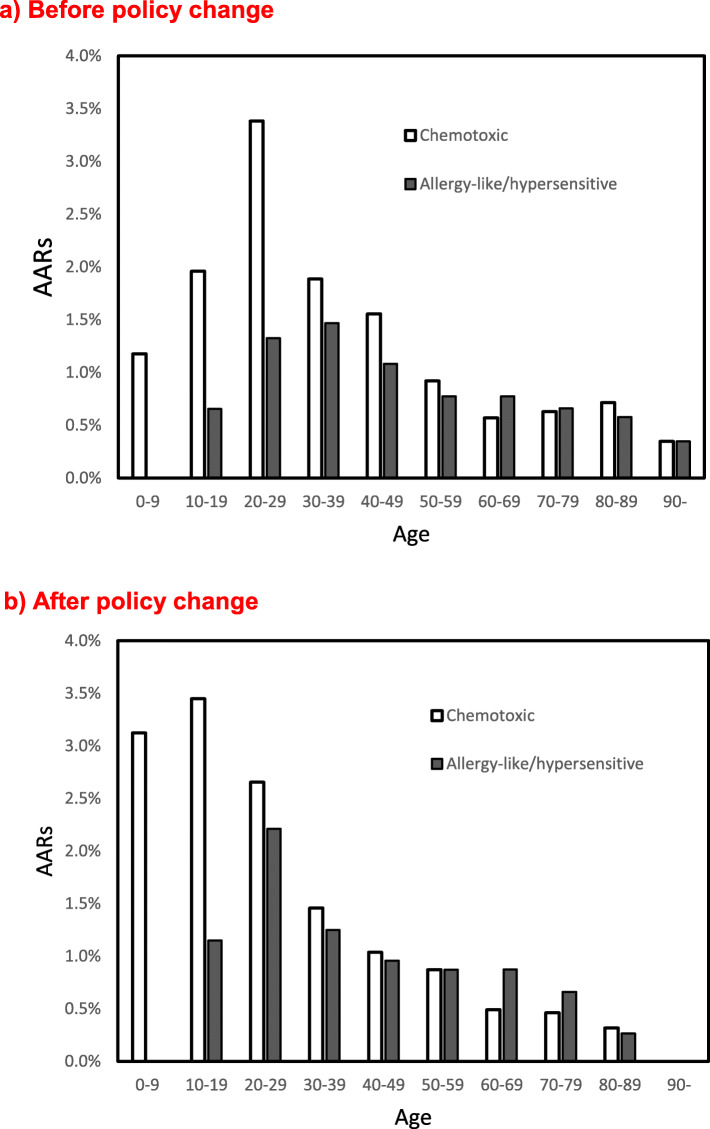
Incidence of adverse reactions by age. **a** Before the policy change. **b** After the policy change

For chemotoxic reactions, the incidence of nausea significantly decreased (0.31 to 0.18%, *p* = 0.006) after the policy change, but the incidence of vomiting did not change (0.12 to 0.16%; Table 2). Both chemotoxic and allergy-like/hypersensitive reactions tended to decrease with age (*p* < 0.05, Fig. 2) both before and after the policy change. No cases of aspiration pneumonia attributable to vomiting were found in the patients’ chart review either before or after the policy change.

There was no significant change in the incidence of severe hypersensitivity/allergy-like reactions (hypotensive shock) before and after the policy change (0.06 to 0.05%). There were no fatal cases.

## Discussion

In this study, no increase in the overall incidence of AARs, including severe allergy-like/hypersensitive reactions, was observed after the policy change. No increase in vomiting was observed, and no cases of aspiration nor aspiration pneumonia were found on retrospective chart review.

Li et al. [2] evaluated 110,836 patients and found no significant difference in the incidence of nausea and vomiting between the fasting and non-fasting groups, with no aspiration symptoms in any cases. Their results were almost consistent with our results. Their study did not state differences (or lack thereof) in incidences of other types of AARs [2]. Our results were also consistent with those for patients prior to percutaneous coronary intervention (PCI; *n* = 1916), in which there were no cases of intraprocedural or postprocedural aspiration pneumonia, despite none of the patients being nil-per-os/nil-by-mouth (NPO/NBM) prior to their coronary procedures [6]. In their study, there were some adverse effects suspected to be related to coronary intervention procedures, but the incidence of AARs suspected of being associated with contrast media was unknown [6]. In the current study, we confirmed that the other types of AARs did not increase and that there were no cases of aspiration pneumonia due to vomiting during both study periods.

Interestingly, in our study, the incidence of nausea was significantly decreased (0.31 to 0.18%) after the policy change. This result may be consistent with the study by Oowaki et al., in which nausea and vomiting occurred more frequently in patients who fasted for longer periods before contrast-enhanced CT [7]. Barbosa et al. [4] evaluated 3206 patients and reported that the incidence of some symptoms, such as flushing, dizziness, ear pruritus, tingling, tremor, pain at the injection site, tachycardia, and headache, was lower in the non-fasting group. The reason for the reduction in the incidence of nausea in our study is unclear for us. However, if patients fast too much, they may not feel well, and nausea can occur as well, or when they are hypoglycemic. Anyway, this lower incidence is certainly beneficial to patients and may be a reason why not to recommend fasting before contrast-enhanced CT. The incidences of both chemotoxic and allergy-like/hypersensitive reactions decreased with age. This result was consistent with a previous study [8]. The reason for this decrease was unclear to the authors as it was to us, but there may be some psychological factors, particularly in patients under 29 years [8].

Another important concern of fasting has been raised by Kim et al. [3]. They evaluated 1175 patients instructed to fast solid food for 6 h prior to contrast-enhanced CT and found that many patients excessively fasted fluid as well as solid food. They concluded that patients should be recommended to exercise caution against dehydration and not to fast excessively.

Instructions to fast for several hours prior to administration of contrast media were considered essential, and this policy has traditionally been kept due to concerns about vomiting and its potential to cause aspiration [1]. However, there has been little evidence to support this practice. Our current study, along with previous studies [2, 6], has confirmed not only the lack of benefit but also the possible disadvantages of fasting prior to contrast-enhanced CT. Preparative fasting should be recommended only before specific types of imaging examinations, such as virtual gastroscopy and colonoscopy, 3D anatomic reconstruction for preoperative planning, and examinations performed under general anesthesia or sedation [4].

There were several limitations in our study. First, in our institution, five types of non-ionic low-osmolar contrast media were used for CT examinations. Most non-ionic contrast media have similar properties, and there have been no reports showing clear differences in the incidence and types of AARs [5]. It is well known that a change of contrast media can cause changes in the AAR rate as Weber effect [5], but during the study period, there was no major change in radiologists’ charge nor change in the CT protocol. We suspect that our findings may be extrapolated to most non-ionic low-osmolar contrast media. Second, this study was conducted only in one institution. However, it is reasonable to expect the incidence of AARs to be similar in institutions with similar policies. Third, in the current study, the patients before the policy change were employed for the so-called historical control. Selection bias could not be completely avoided, but we did not recognize any change in the content or quality of medical care before and after the policy change. Fourth, there was no information about time length of fasting.

In conclusion, abolishing instructions to fast prior to contrast-enhanced CT did not increase AARs of any type. The current study provided no direct evidence suggesting that fasting prior to contrast-enhanced CT prevented vomiting, and no aspiration pneumonia attributable to vomiting was observed. The significantly decreased incidence of nausea may decrease patient discomfort. Our study confirmed that fasting is not required prior to CT examination with non-ionic low-osmolar iodinated contrast media.

## Data Availability

All data was obtained from the database of our institution, based on the approval of the institutional research ethics committee.

## Abbreviations

AARs: Acute adverse reactions
ESUR: The Society of European Urogenital Radiology
